# Expert consensus on practical aspects in the treatment of chronic urticaria

**DOI:** 10.1007/s40629-021-00162-w

**Published:** 2021-02-24

**Authors:** Andrea Bauer, Heinrich Dickel, Thilo Jakob, Andreas Kleinheinz, Undine Lippert, Martin Metz, Sibylle Schliemann, Uwe Schwichtenberg, Petra Staubach, Eva Valesky, Nicola Wagner, Bettina Wedi, Marcus Maurer

**Affiliations:** 1 University Hospital Carl Gustav Carus, Department of Dermatology, University Allergy Center, Urticaria Center of Reference and Excellence (UCARE), Technical University, Dresden, Germany; 2 Department of Dermatology, Venereology and Allergology, St. Josef Hospital, Ruhr University Bochum, Bochum, Germany; 3 University Medical Center Giessen (UKGM), Department of Dermatology and Allergy, Justus-Liebig-University Giessen, Giessen, Germany; 4 Department of Dermatology, Urticaria Center of Reference and Excellence (UCARE), Elbe Medical Centre, Buxtehude, Germany; 5 Department of Dermatology and Allergology, Urticaria Center of Reference and Excellence (UCARE), University Medical Center Göttingen, Göttingen, Germany; 6 Department of Dermatology and Allergy, Urticaria Center of Reference and Excellence (UCARE), Charité—Universitätsmedizin Berlin, Charitéplatz 1, 10117 Berlin, Germany; 7 Department of Dermatology, Urticaria Center of Reference and Excellence (UCARE), University Hospital Jena, Jena, Germany; 8 Derma-nord dermatologists Dr. Schwichtenberg, Bremen, Germany; 9 Department of Dermatology, Urticaria Center of Reference and Excellence (UCARE), University Medical Center Mainz, Mainz, Germany; 10 University Hospital Frankfurt, Department of Dermatology, Venerology and Allergology, Goethe University, Frankfurt, Germany; 11 Department of Dermatology, Urticaria Center of Reference and Excellence (UCARE), University Medical Center Erlangen, Erlangen, Germany; 12 Department of Dermatology and Allergy, Urticaria Center of Reference and Excellence (UCARE), Comprehensive Allergy Center, Hannover Medical School, Hannover, Germany

**Keywords:** Treatment recommendations, H1 antihistamines, Omalizumab, Diagnostics, Therapy

## Abstract

**Background:**

Chronic urticaria (CU) is a common disease which represents a considerable burden for many patients. The current urticaria guideline describes the evidence-based diagnosis and treatment of CU. In addition, however, questions often arise in everyday practice that are not addressed by the guideline.

**Methods:**

In May 2020, a digital meeting with German urticaria experts was held, in which practical aspects of CU treatment were discussed and supporting aids for everyday clinical treatment formulated. The resulting advice in this document focus on practical questions and the available literature and experiences of the participants.

**Results:**

The diagnosis of CU can be made in a short time by means of a thorough anamnesis, a physical examination, and a basic laboratory chemical diagnosis. For this purpose, practical recommendations for everyday practice are given in this paper. An extended diagnosis is only indicated in a few cases and should always be carried out in parallel with an effective therapy. In general, CU should always be treated in the same way, regardless of whether wheals, angioedema or both occur. Symptomatic therapy should be carried out according to the treatment steps recommended by the guidelines. This publication provides practical advice on issues in everyday practice, such as the procedure in the current coronavirus disease 2019 (COVID-19) pandemic, the cardiac risk under higher dosed H1 antihistamines, the self-administration of omalizumab as well as vaccination under omalizumab therapy. In addition to treatment recommendations, topics such as documentation in the practice and family planning with urticaria will be discussed.

**Discussion:**

These supporting treatment recommendations serve as an addendum to the current CU guideline and provide support in dealing with CU patients in everyday practice. The aim is to ensure that patients suffering from CU achieve complete freedom of symptoms with the help of an optimal therapy.

**Supplementary Information:**

The online version of this article (10.1007/s40629-021-00162-w) contains supplementary material, which is available to authorized users.

## Introduction

Chronic urticaria (CU; duration of symptoms longer than 6 weeks) is a common disease associated with a considerable burden for many patients, as itching, wheals and angioedema are often not sufficiently controlled. The disease, which occurs at any age, is divided into chronic spontaneous (i.e. with spontaneous onset of symptoms) urticaria (csU) and chronic inducible (i.e. with symptoms triggered by reproducible specific triggers such as cold or pressure) urticaria (CIndU) [1]. Hybrid types (i.e. symptomatic dermographism plus csU) can also occur. The effects of CU go beyond the physical symptoms and significantly reduce the quality of life of those affected. In one third of patients, CU is associated with depression, anxiety as well as sleep disorders, and patients’ performance in everyday life, at school and at work is often significantly impaired [1–3]. Therefore, the treatment goal is to achieve complete freedom of symptoms. For many patients, this goal can be achieved with the currently available treatment options. However, these are frequently not optimally implemented in clinical practices. In many cases, drug treatment in everyday clinical practice does not correspond to the guideline recommendations [4]. Current data from the AWARE study show that, despite therapy, about one third of patients do not achieve adequate disease control even after two years [5]. General practitioners are often the initial contacts. In the case of mild disease courses, H1 antihistamine treatment is usually sufficient. In the case of therapy resistance, a referral to a specialist should be made for escalation of therapy in accordance with the guidelines, and if necessary, also for further diagnosis. The current urticaria guideline discusses in detail the evidence-based diagnosis and treatment of CU. But beyond that, questions often arise in clinical everyday life. For this reason, a meeting of German urticaria experts was held in May 2020 with the aim of discussing practice-relevant aspects of CU treatment and formulating supporting aids for everyday clinical treatment. These resulting pieces of advice serve as a supplement to the current CU guidelines. They focus on practical issues and are based on the available literature as well as experience of the experts in this field.

## Chronic urticaria in times of COVID-19

To date, there is no evidence that patients with CU have a higher risk of severe COVID-19 due to their disease. According to current knowledge, treatment with H1 antihistamines and biologicals such as omalizumab does not represent an additional risk factor. Studies even suggest that omalizumab can reduce virus-mediated exacerbations [6, 7], which could possibly be beneficial in case of a SARS-CoV‑2 (severe acute respiratory syndrome coronavirus type 2) infection [8].

It is important to achieve adequate symptom control of CU, even in times of the COVID-19 (coronavirus disease 2019) pandemic. National and international allergology societies and associations (AeDA, DGAKI, GPA, LGAI, ÖGP, ARIA, EAACI^1^) therefore recommend that biological therapy should be continued unchanged in patients without suspected or proven SARS-CoV‑2 infection. Patients with a mild/moderate COVID-19 course are also recommended to continue therapy (under risk–benefit assessment and with the patient’s consent). In case of severe disease, an extension of the interval or interruption of therapy may be considered [9].

## Diagnosis of csU

The objectives of the diagnostic evaluation of patients with csU comprise the exclusion of other diseases (differential diagnosis), testing for trigger factors relevant to csU (e.g. taking painkillers), evaluating comorbidities, and determining disease activity (symptoms), impairment of quality of life as well as disease control.

### Anamnesis and basic diagnostics

The diagnosis begins with a detailed anamnesis that should be focused on central information. The guideline, which contains 13 specific questions, can be helpful during this process [10]. It is recommended to use a standardised anamnesis questionnaire. A basic questionnaire (see appendix) or the more detailed patient questionnaires of the Chronic Urticaria Registry (CURE) are suitable for initial and follow-up visits (available at: http://www.urticaria-registry.com/for-participants.html).

Following the anamnesis (including questions on atopic diathesis and gastrointestinal symptoms), a physical examination and basic laboratory diagnostics should be performed. The basic diagnostic tests include the determination of the blood sedimentation rate (BSG) and/or the C‑reactive protein (CRP) and a differential blood count [10].

If the anamnesis provides a specific indication for extended diagnostic tests, an effective guideline-based therapy with the aim of complete freedom of symptoms should immediately be started in parallel to the initiated examinations.

### Differential diagnostics

Important differential diagnoses in which wheals and angioedema can also occur (e.g. autoinflammatory diseases) should be considered in the anamnesis by means of specific queries (see points in Infobox 1). If a suspicion arises, further clarification by a specialist is recommended.

#### Infobox 1

1. Family history of wheals and angioedema

– A positive family history may indicate congenital diseases such as autoinflammatory syndromes and hereditary angioedema (HAE).

2. Timing of disease onset

– Onset in early childhood may indicate congenital conditions such as cryopyrin-associated periodic syndrome (CAPS) or HAE.

3. Occurrence of angioedema without the presence of wheals

– Isolated occurrence of angioedema may indicate bradykinin-mediated angioedema, e.g. HAE or ACE inhibitor/sartan-mediated angioedema.

4. Duration and location of angioedema

– Angioedema that persist for several days, angioedema of the larynx or abdomen or angioedema that do not respond to glucocorticoid therapy may indicate HAE or other bradykinin-mediated angioedema.

5. Duration and consequences of wheals

– Long duration of wheals (usually > 24 h) or subsequent haematoma or hyperpigmentation may indicate urticarial vasculitis.

6. Medication

– Taking ACE inhibitor or sartan may indicate ACE inhibitor/sartan-mediated angioedema.

7. Associated symptoms

– Bone/joint pain, signs of inflammation or fever may indicate autoinflammatory syndrome.

8. Occurrence of wheals and/or angioedema depending on specific triggers

– The exclusive occurrence of wheals and/or angioedema due to specific triggers (e.g. skin exposed to cold) indicates chronic inducible urticaria.

9. Previous therapy and response to therapy (including dosage and duration)

– Therapy resistance may indicate autoinflammatory syndrome or bradykinin-mediated angioedema.

10. Previous diagnostic procedures/results

11. Intermittent occurrence of urticaria and/or accompanying symptoms, e.g. gastrointestinal problems

– May indicate an IgE-mediated food allergy.

**Fig. 1:**
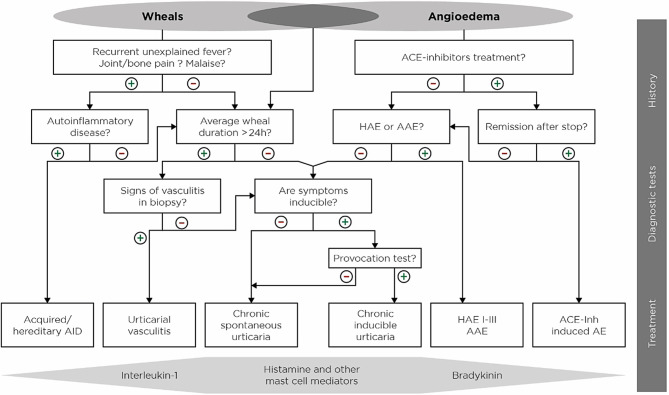
Diagnostic algorithm for patients with wheals and/or angioedema. *AAE* acquired angioedema due to C1-inhibitor deficiency, *ACE-Inh* angiotensin-converting enzyme inhibitor, *AE* angioedema, *AID* auto-inflammatory disease, *HAE* hereditary angioedema [10]

In patients with wheals (but no angioedema), urticarial vasculitis and autoinflammatory diseases such as Schnitzler syndrome or cryopyrin-associated periodic syndrome (CAPS) must be excluded. For differential diagnosis, the use of the diagnostic algorithm of the guidelines is recommended (Fig. 1; [10]).

Angioedema are often underdiagnosed in patients with csU. They have a negative impact on quality of life and daily activities [11]. Approximately 10% of all patients with csU experience angioedema only without wheals. In these csU patients with isolated recurrent angioedema, there is a risk of confusion with bradykinin-mediated forms of angioedema, where wheals are also not present. It is therefore relevant for the initiation of therapy to correctly diagnose and classify angioedema. Bradykinin-mediated angioedema include angiotensin-converting enzyme (ACE)-inhibitor-induced angioedema, sartan-mediated angioedema, and hereditary angioedema (HAE) as well as angioedema due to acquired C1 inhibitor deficiency. These differential diagnoses can be proven or ruled out by a specific anamnesis, discontinuation of suspected medication (ACE inhibitors) and laboratory tests (determination of the concentration as well as activity of the C1 esterase inhibitor [C1-INH] and the complement factor C4 and possibly C1q). It should be emphasised that csU-associated angioedema rarely lead to swelling of the tongue and only in exceptional cases affect the larynx (e.g. non-steroidal anti-inflammatory drugs [NSAIDs] as a pharmacological trigger). Patients should be informed about this to reduce any anxiety.

In addition, differentiation from other cutaneous/subcutaneous swellings is essential (see Infobox 2 for examples).

#### Infobox 2

- Granulomatous inflammation (e.g. cheilitis granulomatosis, genital oedema in Crohn’s disease)
- Contact urticaria, acute allergic contact eczema
- Air emphysema (e.g. after fractures, endodontological treatments)
- Tumours
- Oedema in heart failure or chronic venous insufficiency
- Pregnancy oedema and oedema in premenstrual syndrome

In few cases, a true IgE-mediated food allergy may manifest itself under the clinical picture of urticaria. Diagnosis of this should be carried out by a specialist/allergist. Intermittent occurrence of urticaria and/or accompanying symptoms, such as gastrointestinal problems, may be indicative. This form of food allergy is rarely relevant in patients with CU but is more likely if wheals do not occur daily. Pseudo-allergic reactions (non-IgE-mediated hypersensitivity) to various food ingredients and additives are more frequently observed.

### Evaluation of triggering factors

Since there are variable triggering factors of csU, the medical history is of central importance in identifying triggers, but also predictors of disease progression and therapy as well as factors influencing disease activity [10]. We advise against a premature search for infections without anamnestic signs; however, this should be done in cases of prolonged persistence and/or severe suffering.

### Evaluation of comorbidities

In routine diagnostics, frequent comorbidities of csU should be clarified by anamnesis and, if necessary, further diagnostic tests. Comorbidities can influence the severity of csU, the impairment of quality of life and the response to therapy.

#### Autoimmune diseases (especially of the thyroid gland)

The determination of thyroid hormones and autoantibodies to identify comorbidity (Hashimoto’s thyroiditis) is recommended, as patients with csU are more often affected by autoimmune thyroid diseases [12].

#### Psychiatric comorbidities

Patients with csU often develop psychiatric comorbidities such as depression and anxiety disorder [13]. For these patients, interdisciplinary cooperation and therapy, e.g. with psychotherapists, is recommended.

#### CIndU

CIndU often occurs as a simultaneous disease in csU (about 20%) and is the most common comorbidity in H1-antihistamine-resistant csU [40]. It should therefore always be examined whether CU patients also suffer from CIndU and vice versa.

### Assessment of disease activity, impairment of quality of life and disease control

In everyday clinical practice, the assessment of the disease burden, defined as disease activity plus quality of life and disease control, supports the decision whether therapy is successful or should be escalated if necessary.

To monitor disease activity, the weekly urticaria activity score (UAS7) [14] and the angioedema activity score (AAS) [15] are used. To calculate the UAS7, wheals and itching are documented and quantified daily by the patient in a diary over seven consecutive days [14]. The occurrence of angioedema is recorded along the same lines with the AAS [15].

It is important to note that the UAS7 is only applicable to csU and not suitable for CIndU. However, CIndU-specific activity scores are available (CholUAS7 for cholinergic urticaria) or in development (ColdUAS for cold urticaria, SDAS for symptomatic dermographism).

To assess disease control and therapy success in csU, CIndU, and combinations of both subtypes, the urticaria control test (UCT) is used. With four simple questions, it enables a quick and reliable assessment of the disease situation during the last four weeks. Each of the four UCT questions is answered by the patient, and the resulting scores (0–4 per answer) are added to a total score of 0–16. The threshold for controlled urticaria is reached at 12 points. Uncontrolled urticaria is indicated if the patient reaches ≤ 11 points [16].

In addition, regular assessment of the quality of life is useful to estimate treatment success. In addition to the DLQI, two instruments are available for this purpose: the Chronic Urticaria Quality of Life Questionnaire (CU-Q2oL) [17] and the Angioedema Quality of Life Questionnaire (AE-QoL) [18].

## Therapy of CU

The primary and uniform goal of CU treatment is to achieve complete freedom of symptoms (treat the disease until it is gone). The therapeutic approach consists of two aspects:

- Elimination and prevention of relevant triggers and
- Symptomatic drug therapy [10].

### Elimination and prevention of relevant triggers

If a patient has one or more relevant triggers for the occurrence of wheals and/or angioedema (often there are no known triggers), these should be eliminated or avoided if possible. Avoidance almost never results in complete recovery, but it can lead to an improvement of symptoms. Drugs suspected of being triggers should be discontinued or, if necessary, replaced with drugs from another substance class. If csU is triggered by NSAIDs, a switch of analgesics to paracetamol can be attempted. From a differential diagnosis point of view, a NSAID hypersensitivity must be considered.

If physical stimuli trigger the disease, avoidance is desirable, but often not feasible in everyday life. Here it is important to provide patients with knowledge about their illness. In the case of pressure urticaria and symptomatic dermographism, for example, it should be explained that even simple methods (e.g. wider pocket straps, avoidance of tight-fitting clothes and belts) can help to reduce the development of symptoms. As it is not always possible to avoid cold when suffering from cold urticaria, patients should also be offered help in everyday life. Thick, warm clothing, (ski) gloves and warm shoes/socks are important protective measures against the cold. Unprotected areas such as the face should be treated with a fatty ointment before going outside. In case of light urticaria, the triggering wavelength range should be determined by means of “light stairs”. In patients whose urticaria reacts to ultraviolet (UV) light, UV protection and/or UV hardening can reduce the development of urticarial complaints.

If pseudo allergy is suspected and daily symptoms are present, it is recommended that a low-pseudo allergen diet is followed for at least two to three and a maximum of four weeks. If gastrointestinal symptoms are also present, a low-histamine diet may also be advisable [19]. As yet, however, there is little evidence of the effect of diets on urticaria symptoms [10].

## Guideline-based therapy with H1 antihistamines (therapy levels 1 and 2)

**Fig. 2:**
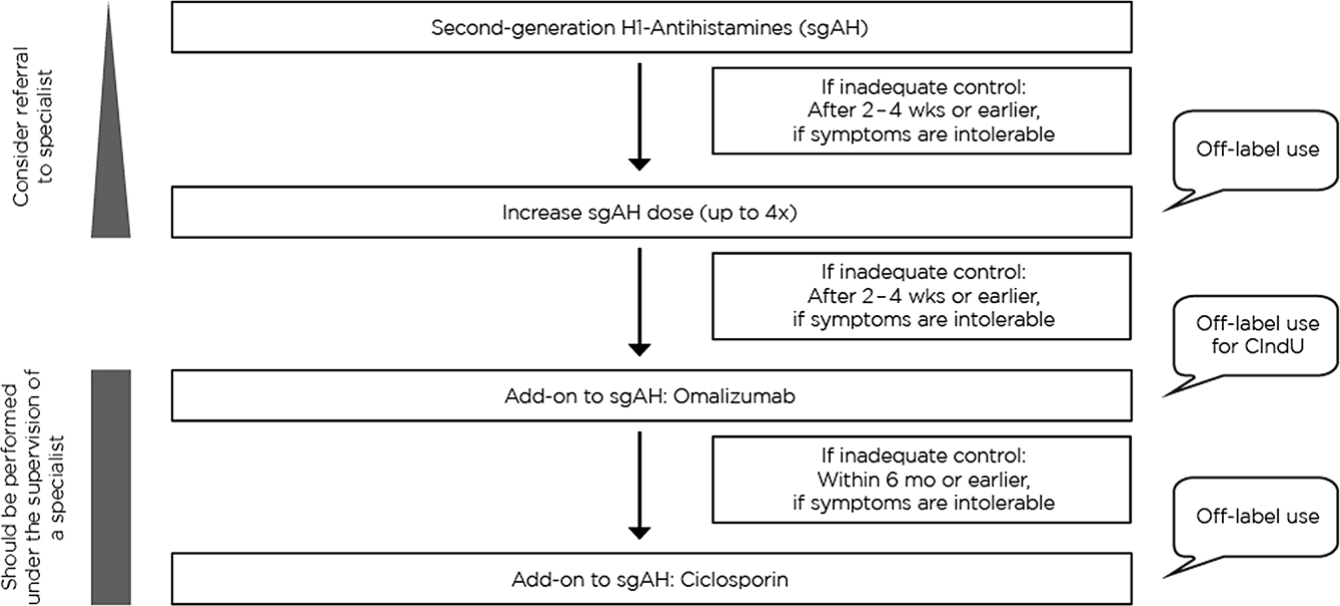
Recommended therapy algorithm for the treatment of chronic urticaria. *wks* weeks, *mo* months, *CIndU* chronic inducible urticaria

CU should always be treated in the same way, regardless of whether wheals, angioedema or both occur. Thus, the therapy of CIndU is equivalent to therapy of csU. Symptomatic therapy should be carried out according to the levels scheme recommended by the guidelines (Fig. 2; [10]).

A second-generation non-sedating H1 antihistamine is the first treatment of choice. If continuous intake over two to four weeks does not lead to adequate control of symptoms, the guideline recommends a higher dosage up to four times of standard dosage. This higher dosage is often more effective than the standard dosage [20], but is off-label use.

### Type of higher dosing

In this situation, 2–0–2 is the preferred treatment regimen, alternatively one tablet can be taken four times a day; however, this requires very good compliance of the patient. It is not recommended to combine different H1-antihistamines.

### Risk of sedation

In case of higher dosing, second-generation H1 antihistamines without significant influence on road safety should be used in the approved daily dose (Table 1). Individual tolerance as well as a possible increase in sedative effects at higher doses should be considered. The patient should be informed about off-label use and possible sedation. In certain situations, e.g. in road traffic or passenger transport, there may be contraindications for off-label dosages.

**Table 1:** Road safety categorisation of H1 antihistamine drugs based on blood alcohol equivalent doses [21, 22]

Generation H1 antihistamines	I No significant effect (DE to BAC: < 0.5 g/l; < 0.5 per mil)	II Low or moderate effect (DE to BAC: 0.5–0.8 g/l; 0.5–0.8 per mil)	III Strong effect, potentially dangerous (DE to BAC: > 0.8 per mil)
1st Generation	–	Ketotifen	Clemastine, diphenhydramine, promethazine
2nd Generation	Azelastine, bilastine, desloratadine, ebastine, fexofenadine, levocetirizine, loratadine, rupatadine	Cetirizine, mizolastine	–

per mil is defined as parts per thousand

*BAC* blood alcohol content, *DE* dose equivalent

### Cardiac risks

Second-generation H1 antihistamines are generally well tolerated. A recent publication shows that they do not exhibit any signs of cardiotoxicity, even though higher doses of up to four times the standard dose are used (including bilastine and rupatadine) [23]. Therefore, in most patients, a higher dose of the standard dose is unproblematic. Possible risk factors include a hereditary long QT syndrome, the simultaneous intake of drugs that can prolong the QT time, or pre-existing heart diseases (e.g. bradycardia). These should be excluded beforehand.

## Guideline-based additional therapy with omalizumab (therapy level 3)

If there is no sufficient improvement after two to four weeks of therapy with a higher-than-standard dose of second-generation H1 antihistamine, treatment with approved IgE antibodies such as omalizumab should additionally be administered (Fig. 2). The same recommendations apply to children and older patients.

Omalizumab is approved for the treatment of csU from the age of 12 years but may also be considered in younger patients. Case reports in children under 12 years of age indicate that efficacy and safety do not differ between paediatric and adult patients [24, 25]. For the treatment of severe allergic asthma, omalizumab is approved in children as young as 6 years of age and the safety data show that it is well tolerated [26], so that off-label use in children as young as 6 years of age can be considered. This must, however, be discussed accordingly with the parents or guardians and is the sole responsibility of the attending physician.

Omalizumab treatment is also described as effective and safe in patients with CIndU [39], but it is not approved for this indication and therefore represents an individual treatment use. This applies to patients who only suffer from an inducible form and not also from csU. For patients who suffer from both CIndU and csU, therapy with omalizumab is within the label. When documenting the prescription of omalizumab, it is therefore important to ensure that both forms of CU (CIndU and csU) are well documented.

### Dosage and interval

The approved dose of omalizumab in Europe is 300 mg every four weeks as a subcutaneous injection. Other doses and interval changes have been studied [27, 28], but are not approved. In individual cases of a partial response only, an increase in dosage or a reduction of the interval can be considered. If the response is very good, a dose reduction or an extension of the interval may be eligible [29]. Any deviation from the approved scheme, however, is considered off-label use.

### Safety

#### Anaphylaxis

The results of clinical trials show that omalizumab has a good safety profile [30]. It is important to note that omalizumab is a biological with a low rate of anaphylaxis (0.2%). This has been shown in both, pivotal clinical trials [31–33] as well as post-marketing analyses [34, 35]. Most anaphylaxis occurred within 2 h of injection during the first three applications [35]. Patients with anaphylaxis in their medical history [36] due to food, drugs (especially biologicals), vaccinations, polysorbate or insect venom are at higher risk for anaphylaxis. Patients should be educated about possible symptoms of anaphylactic shock (skin reactions, effects on the respiratory tract and cardiovascular system). However, it should also be pointed out that in the case of mild reactions (e.g. increased wheals), injections can be continued without elevated anaphylaxis risk.

#### Malignancies

In the phase I–III clinical trials from the asthma study programme, a numerical, but not significant imbalance in malignancies was observed between malignant tumours in patients treated with omalizumab and control patients. A causal relationship between medication and malignant neoplasms was considered unlikely due to the variety of tumour entities and the relatively short exposure [37]. Long-term data from the EXELS follow-up study show no differences regarding malignancy risk between patients with or without omalizumab therapy [38] and a pooled data analysis of 32 randomised, double-blind, placebo-controlled studies also found no association between omalizumab and an increased risk of malignancy [39]. Patients with active malignant tumours must be individually assessed for the risk/benefit of using omalizumab. There is no absolute contraindication.

#### Combination with other biologicals

Biologics have significantly expanded the therapeutic options for various diseases in recent years, so that their use in clinical practice is increasing. It is therefore possible that CU patients may receive another biological in addition to omalizumab due to another disease (e.g. atopic dermatitis or rheumatoid arthritis). The data situation regarding the safety and efficacy of various combinations of biologicals has so far not been sufficiently investigated. However, based on case reports and our own experience, no safety risks have been identified to date in combining omalizumab with other biologicals, such as mepolizumab, benralizumab, dupilumab or etanercept [40–43].

#### Infections

Infections can be triggering factors of CU. In clinical practice, the question frequently arises whether omalizumab can be used without risk in patients with infections. Pragmatically, pausing omalizumab in the case of febrile infections or systemic need for treatment with antibiotics until complete healing can be useful. In cases of a mild cold, cough, or hoarseness, omalizumab can be administered without hesitation.

### Practical aspects

#### Vaccinations under omalizumab

Attenuated live vaccines as well as inactivated vaccines provide protection by neutralising IgG antibodies produced by B lymphocytes. No impairment of this pathway by the anti-IgE antibody omalizumab is known. As the mechanism of action of omalizumab does not cause immunosuppression, there is no contraindication to live vaccines (e.g. measles, mumps, rubella). Patients undergoing omalizumab therapy should therefore not be deprived of vaccinations. An interval of at least one week between an injection of omalizumab and plannable vaccinations is recommended. Immediate necessary vaccinations (e.g. tetanus) can be given at any time.

#### Self-administration

Patients without a history of anaphylaxis may inject omalizumab themselves or have a caregiver inject it from the fourth use onwards if a doctor considers it appropriate. Treatment should be given for an initial period of six months, followed by a review of disease activity. If the patient fears the occurrence of anaphylaxis, the administration should be carried out again in the practice. Risk patients with anaphylactic reactions in their medical history and pregnant women are excluded from the possibility of self-administration. If patients relapse after discontinuation of omalizumab and treatment with omalizumab is renewed, this can also be done by self-administration.

Before starting the administration, the skin should be disinfected. The recommended place for self-administration is the subcutis of the abdominal wall or the side of the thigh extensor. If the injection is carried out by a caregiver, it can be injected subcutaneously into the upper arm. The individual steps of the administration as well as important information on transport and storage can be found on the package insert. Patients should be made aware that in case of intending to travel, a multilingual medical certificate confirming the necessity of carrying omalizumab in their hand luggage is required, while observing the storage conditions (e.g. on-board refrigerator or medicine cooler).

## Guideline-based additional therapy with ciclosporin A (therapy level 4)

If there is no therapeutic success after six months of treatment with omalizumab, the guidelines recommend off-label use with ciclosporin A (CSA) in addition to existing therapy with H1 antihistamines. Recommended dosages are 4 mg/kg or less [44]. Experience shows that a therapeutic response occurs within four to eight weeks. Since adverse effects can occur more frequently during therapy, this treatment option should be reserved for therapy-resistant cases and a careful monitoring of side effects should be carried out:

- *Questioning and clinical examination*: hypertrichosis, gingival hyperplasia, blood pressure control, tremor, paraesthesia, gastrointestinal complaints.
- *Laboratory tests*: BSG, CRP, blood count including platelets, alkaline phosphatase, alanine aminotransferase, creatinine, potassium, urine test strip.

CSA should not be used in patients with impaired kidney function, untreated hypertension, untreated infections, or any form of malignancy. The patient should be informed about the off-label use of CSA, as well as the control examinations and possible side effects. Information sheets for patients and doctors can be downloaded (in German) from the website of the German Society for Rheumatology (https://dgrh.de/Start/Versorgung/Therapieinformationen/Therapieinformationsbögen.html).

## Systemic glucocorticoids

In case of acute exacerbations, treatment with sufficient doses of oral systemic glucocorticoids can be given for a short period (up to a maximum of ten days) to reduce the duration and activity of the disease. A medium-high dose of prednisolone of 20–50 mg/day for a maximum of ten days is recommended. There is usually no need to taper the medications if used for three to five days [10, 44]. Long-term treatment with systemic glucocorticoids should be avoided at all costs due to the high rate of side effects.

## Documentation of therapy in the clinical practice

The therapeutic goal in the treatment of CU is to achieve complete freedom of symptoms. With the therapy options available today, this goal can be achieved for many patients. However, not all options/doses are within the label, so that good documentation of the guideline-based therapy is of high relevance, also with regard to off-label use not being tolerated by pharmaceutical companies. The documentation must show that the CU is a serious and debilitating disease with lasting effects. This is sometimes questioned by the payers. In the best case, quality of life questionnaires should be used. If this is not possible in terms of time, anamnesis entries can also be made that reflect the severity of the suffering.

Many patients can be adequately treated with nonprescription H1 antihistamines. At the expense of the statutory health insurance system, these drugs for adults can only be prescribed in exceptional cases for very severe forms of CU or severe, long-lasting pruritus, which must be documented in the patient’s medical file. Non-sedative antihistamines can be prescribed for patients insured by public health insurance if nonprescription second-generation H1 antihistamines (loratadine, desloratadine, cetirizine, levocetirizine) were proven to be ineffective or incompatible. However, this is rare and must be documented in the patient’s medical file. Exceptions to this rule apply for example to children up to the age of 12. If an over-the-counter (OTC) therapy is not listed in the medical file, as OTC prescriptions do not have to appear there, another form of proof that such a therapy attempt has been made is required.

If the standard dose of second-generation H1 antihistamines does not lead to symptom control, a subsequent fourfold dosage of second-generation H1 antihistamines follows the guidelines and is safe according to current knowledge. However, patients should be advised that higher doses of second-generation H1 antihistamines as well as treatment with CSA are equivalent to off-label use. The corresponding risk disclosure and the information of possible non-reimbursement by statutory health insurances must be carefully documented and should best be countersigned by the patient. Privately insured patients should ask their health insurance company about a possible reimbursement before starting therapy. Lack of therapy successes should best be documented with scores. The UCT can be carried out in an outpatient setting with little effort. The UAS7 in the form of an urticaria diary can also be very helpful in difficult disease courses.

The csU is not uniquely codable, but the coding can be supplemented via a text box within the software. Important codes for patients with urticaria are shown in Table 2.

**Table 2:** Important ICD-10 codes

	ICD-10 Code	Description	Practical implementation
Urticaria	L50.1	Idiopathic urticaria	Acute urticaria
	L50.2–.6 + L56.3	Inducible urticaria (per cause)	Inducible urticaria (coldness, warmth, etc.)
	L50.8	*Other urticaria* Chronic Recurrent, periodic	To be preferred as coding for chronic spontaneous urticaria
	L50.9	Urticaria	Not recommended for coding
Angioedema	T78.3	Angioneurotic oedema/Quincke’s oedema	–
Pruritus	L29.8	Other pruritus	–
	L29.9	Pruritus not specified	–
Additional codes	Z72.8	Highly debilitating living situation	–
	Z51.88	System therapy	–
	T88.8	Only slight improvement in symptoms	–

The current ICD-10 classification is available at https://www.dimdi.de/static/de/klassifikationen/icd/icd-10-gm/kode-suche/htmlgm2020/

*ICD* International classification of diseases

## Urticaria and family planning

In general, patients with CU can have children. Therapy options are available that can also be used during pregnancy. The therapeutic goal of freedom of symptoms also applies during pregnancy. Pregnant women with uncontrolled CU should be referred to a specialised centre.

Although H antihistamines are not approved for use during pregnancy, experience (especially with loratadine and cetirizine) does not indicate an increased risk of malformation. These two substances can preferably be given in a once daily dosage (https://www.embryotox.de/). The desire to have children is not a reason to stop or omit therapy with loratadine or cetirizine if this results in good symptom control of CU. First-generation H1 antihistamines, on the other hand, are not recommended even at night due to placental passage and the disruption of REM (rapid eye movement) sleep. There have been no studies on the safety of up dosing of second-generation H1 antihistamines in pregnant women.

For omalizumab, the EXPECT pregnancy registry found no evidence of a pregnancy-induced risk of congenital anomalies or thrombocytopenia in patients with asthma (230 documented pregnancies) [45]. The guideline also describes the use of omalizumab in pregnancy as safe [10]. If there is a clinical need, which may be caused by the patient’s exposure to uncontrolled CU including sleep loss, the use of omalizumab during pregnancy may be considered after appropriate risk assessment and patient education. However, self-administration is not recommended for these patients.

## Summary and conclusion for clinical practice

- With the aid of thorough anamnesis, physical examination, and basic laboratory diagnostics, a diagnosis can be made in a short time.
- For the anamnesis, a short basic form or the more detailed CURE patient forms for initial and follow-up presentation are suitable.
- An extended diagnostic evaluation is only indicated in a few cases and should always be carried out in parallel with an effective therapy.
- Angioedema are often underdiagnosed in patients with csU, although they can be part of csU regardless of the presence of wheals and can have a negative impact on quality of life and daily activities.
- A csU is therefore always treated in the same way, independent of the presence of wheals angioedema, or both.
- The treatment of choice for csU is a second-generation H1 antihistamine. Higher doses, up to a maximum of four times the standard dose, are often more effective but are off-label use and are not tolerated by all patients. In most patients, however, a higher dosage of the standard dose is unproblematic if potential risk factors or comedication are considered (no signs of cardiotoxicity).
- If there is no adequate improvement after 2–4 weeks with a second-generation H1 antihistamine in the standard dosage or, if necessary, after higher dosages, patients with csU should additionally be given omalizumab.
- The therapy of CIndU is equivalent to the therapy of csU. For patients who suffer from both CIndU and csU, therapy with omalizumab is within the label.
- Omalizumab has a good safety profile with a low anaphylaxis rate.
- In cases of mild rhinitis, cough, or hoarseness, omalizumab can be administered without concern.
- Both inactivated and live vaccines can be administered under omalizumab therapy.
- Patients with no known anaphylaxis in their medical history can inject omalizumab themselves or have a caregiver inject it from the fourth application onwards (except pregnant women).
- If there is no therapeutic success after 6 months of treatment with omalizumab, off-label use with CSA in addition to existing therapy with H1 antihistamines is recommended by the guidelines instead.
- In cases of acute exacerbations, treatment with medium-dose oral systemic glucocorticoids can be given for a short period (up to 10 days maximum) to reduce disease duration and activity.
- If clinically necessary, the use of second-generation H1 antihistamines (loratadine, cetirizine) and omalizumab during pregnancy may be considered (off-label).
- Good documentation and education of patients about off-label use is of great importance (the responsibility lies with the treating physician).

## Supplementary Information

Basic questionnaire on nettle rash (urticaria)

## Abbreviations

AAE: Acquired angioedema due to C1-inhibitor deficiency
AAS: Angioedema activity score
ACE: Angiotensin-converting enzyme
ACE-Inh: Angiotensin-converting enzyme inhibitor
AE: Angioedema
AE-QoL: Angioedema Quality of Life Questionnaire
AID: Auto-inflammatory disease
BAC: Blood alcohol content
BSG: Blood sedimentation rate
CAPS: Cryopyrin-associated periodic syndrome
CholUAS: CIndU-specific activity score for cholinergic urticaria
CIndU: Chronic inducible urticaria
ColdUAS: CIndU-specific activity score for cold urticaria
CRP: C‑reactive protein
CSA: Ciclosporin A
csU: Chronic spontaneous urticaria
CU: Chronic urticaria
CU-Q2oL: Chronic Urticaria Quality of Life Questionnaire
CURE: Chronic Urticaria Registry
HAE: Hereditary angioedema
NSAID: Non-steroidal anti-inflammatory drug
SDAS: Activity score for symptomatic dermographism
UAS: Urticaria activity score
UCT: Urticaria control test

## Footnotes

1 *AeDA* Ärzteverband Deutscher Allergologen, *DGAKI* Deutsche Gesellschaft für Allergologie und klinische Immunologie, *GPA* Gesellschaft für Pädiatrische Allergologie und Umweltmedizin, *LGAI* Luxemburgische Gesellschaft für Allergologie und Immunologie, *ÖGP* Österreichische Gesellschaft für Pneumologie, *ARIA* Allergic Rhinitis and its Impact on Asthma, *EAACI* Europäischen Akademie für Allergologie und Klinische Immunologie.
