# Supplementary material for: Expert consensus on practical aspects in the treatment of chronic urticaria
Source: Allergo J Int. 2021 Feb 24;30(2):64–75. doi: 10.1007/s40629-021-00162-w (PMC7903036; doi:10.1007/s40629-021-00162-w)
Supplement: Supplementary file 1 — Basic questionnaire on nettle rash (urticaria) [file 40629_2021_162_MOESM1_ESM.pdf]

Basic questionnaire on nettle rash (urticaria)

Name:

Date of birth:

Date:

Dear patient,

You have a suspected case of urticaria. This is accompanied by itchy, reddish swellings of the upper skin (wheals/hives) and/or of the deeper skin and mucous membranes (angioedema). To better understand and treat your condition, it is important for us to know how you describe your symptoms, which triggers you suspect and which treatments you have already undergone and with which level of success. Please answer all questions if possible. Thank you very much!

1. Which symptoms (itching, swelling of the skin and mucous membranes) occur and since when?

____

____

____

2. What is the course of your symptoms and does anyone in the family have similar complaints?

Describe the average duration (minutes/hours/days) of each swelling. Please state how often (e.g. every day/two times a week etc.) and at which sites swellings occur.

____

____

____

3. Are there any symptoms other than the typical swellings on the skin and mucous membranes that occured frequently in recent times?

These include e.g.

☐ Fever ☐ Joint pain ☐ Shortness of breath

☐ Headaches ☐ Gastrointestinal problems

Further:

____

____

4. Are you aware of/do you suspect any triggers for your urticaria and if so, which ones?

____

____

____

5. What medication are you currently taking for urticaria, what dose and how often do you use it and how well does it help?

Preparation:____

Frequency of use: ____

Effectiveness: ☐ 1 ☐ 2 ☐ 3 ☐ 4 ☐ 5 ☐ 6

1 = Very good effectiveness 6 = No effectiveness

6. Which medications do you take regularly or if needed (e.g. for headaches, joint pain or thyroid disorders) in addition to your permanent medication (please enclose medication schedule)?

____

____

____
